# Implementation of Anaphylaxis Management Guidelines: A Register-Based Study

**DOI:** 10.1371/journal.pone.0035778

**Published:** 2012-05-10

**Authors:** Linus Grabenhenrich, Stephanie Hompes, Hannah Gough, Franziska Ruëff, Kathrin Scherer, Claudia Pföhler, Regina Treudler, Vera Mahler, Thomas Hawranek, Katja Nemat, Alice Koehli, Thomas Keil, Margitta Worm

**Affiliations:** 1 Institute of Social Medicine, Epidemiology and Health Economics, Charité University Medical Centre Berlin, Berlin, Germany; 2 Department of Dermatology, Charité University Medical Centre Berlin, Berlin, Germany; 3 Department of Dermatology and Allergology, Ludwig-Maximilian University, München, Germany; 4 Allergy Unit, Department of Dermatology, University Hospital Basel, Basel, Switzerland; 5 Division of Allergy, Department of Dermatology, The Saarland University Hospital, Homburg/Saar, Germany; 6 Allergy Division, Department of Dermatology, Venerology and Allergy, Universität Leipzig, Leipzig, Germany; 7 Allergy Division, Department of Allergy, University Hospital Erlangen, Erlangen, Germany; 8 Allergy Department, Department of Dermatology, Paracelsus Private Medical University Salzburg, Salzburg, Austria; 9 Allergen Laboratory, Department of Pneumology and Allergology, Children’s Hospital, University Hospital Carl Gustav Carus, Dresden, Germany; 10 Division of Allergology, University Children’s Hospital Zurich, Zurich, Switzerland; 11 Paediatric Epidemiology, Institute of Social Medicine, Epidemiology and Health Economics, Charité University Medical Centre Berlin, Berlin, Germany; 12 Anaphylaxis Research, Department of Dermatology, Charité University Medical Centre Berlin, Berlin, Germany; University of Florida, United States of America

## Abstract

**Background:**

Anaphylaxis management guidelines recommend the use of intramuscular adrenaline in severe reactions, complemented by antihistamines and corticoids; secondary prevention includes allergen avoidance and provision of self-applicable first aid drugs. Gaps between recommendations and their implementation have been reported, but only in confined settings. Hence, we analysed nation-wide data on the management of anaphylaxis, evaluating the implementation of guidelines.

**Methods:**

Within the anaphylaxis registry, allergy referral centres across Germany, Austria and Switzerland provided data on severe anaphylaxis cases. Based on patient records, details on reaction circumstances, diagnostic workup and treatment were collected via online questionnaire. Report of anaphylaxis through emergency physicians allowed for validation of registry data.

**Results:**

2114 severe anaphylaxis patients from 58 centres were included. 8% received adrenaline intravenously, 4% intramuscularly; 50% antihistamines, and 51% corticoids. Validation data indicated moderate underreporting of first aid drugs in the Registry. 20% received specific instructions at the time of the reaction; 81% were provided with prophylactic first aid drugs at any time.

**Conclusion:**

There is a distinct discrepancy between current anaphylaxis management guidelines and their implementation. To improve patient care, a revised approach for medical education and training on the management of severe anaphylaxis is warranted.

## Introduction

### Background

Severe anaphylaxis is an acute and life-threatening IgE-mediated hypersensitivity reaction [1], [2]. A particular cause such as insect venom, food items or drugs is traced in two out of three cases [3]. Beyond skin and gastrointestinal symptoms, airway constriction and circulatory collapse can be fatal.

Due to differences in recognition, diagnosis and reporting of anaphylactic reactions [4], estimates of lifetime prevalence range between 0.05% and 2% [5]. As with other allergic diseases, several surveys suggest a rising incidence of anaphylaxis [6], [7].

Primary prevention strategies have not been established yet, stressing the need for defined first aid management. Different practice parameters have been published to guide initial treatment and secondary prevention of anaphylaxis.

### Management Recommendations

International attempts have repeatedly been made to compile available insight on the diagnosis and management of anaphylaxis [8], complemented through a European initiative focussing on children [9]. On a national scale, the Resuscitation Council (from the United Kingdom) has agreed on a widely implemented guideline [10] giving detailed instructions on the use of emergency drugs and other treatment options.

There is expert agreement [11] to apply adrenaline intramuscularly as first line treatment in all potentially life-threatening anaphylactic reactions in the field, despite inconclusive evidence to support this recommendation [12]. On account of tachyarrhythmia side effects, intravenous application is limited to management involving specialised health care providers such as anaesthetists or emergency physicians.

The use of antihistamines and corticoids is subject to controversy as an ancillary option, the latter supposed to be of particular value in asthmatic individuals. There is no support through controlled trials for both treatment options [13], [14].

The mainstay of secondary prevention is patient education focussing on avoidance of known or suspected allergens and early symptom recognition. Self-injectable adrenaline devices as well as oral antihistamines and corticoids are commonly provided to the patient [15].

Besides the lack of data supporting management recommendations, their use has been only sporadically evaluated.

### Current Implementation

A recent systematic review on studies reporting gaps in anaphylaxis management traced a profound discrepancy between guidelines and their implementation in a widespread variety of settings [16]. Lack of knowledge about diagnosis and treatment was identified to hamper correct management on a professional level, leading to infrequent and delayed use of intramuscular adrenaline and failure to prescribe auto-injectors. Patient instruction on avoidance and proper self-application of first aid drugs was often reported to be insufficient. However, studies on anaphylaxis management generally focussed on a subset of treatment options (e.g. adrenaline) and rely on unique settings such as emergency departments or schools only.

### Anaphylaxis Registry

Hence, to provide sound figures for the current implementation of guidelines, we present the first large-scale analysis of initial treatment and secondary prevention data including all ages and settings, from the transnational anaphylaxis registry, covering the general population of Germany, Austria and Switzerland [17]. This will enable us to spot gaps and target interventional strategies and to improve patient care in severe anaphylaxis.

## Methods

### Design and Subjects

The anaphylaxis registry consecutively recorded incident cases of severe anaphylaxis, first occurrence and recurrent disease. It was aimed to obtain well defined, standardized data of affected patients in Germany, Austria and Switzerland.

Following an acute reaction, patients are being referred to specialized outpatient clinics for further allergological evaluation, usually related to dermatology or paediatric departments of tertiary hospitals. 83 centres participating in the anaphylaxis registry were included in this analysis. At these visits, all individuals reporting respiratory or circulatory symptoms in conjunction with the initial reaction, indicating severe anaphylaxis, were invited to participate and asked to give written informed consent, for children by a guardian.

The study was approved by the ethics committee at Charité University Medical Centre Berlin, Germany.

### Measurement

All information was retrieved from patient records, and if available complemented by emergency physicians’ protocol. Data was acquired anonymously, and entered by trained health professionals in allergy centres into an online questionnaire covering symptoms, diagnostic workup, cause, co-morbidities, and treatment details.

Since speculation about the cause of anaphylaxis is known to be misleading, causes were limited to confirmed cases of insect sting, food or drugs only. Grading of severity was based on symptoms recorded, categorized according to [1]. Information on the person having carried out first aid treatment was pooled in 5 clusters to reflect assumed level of training in emergency handling, professionals in 3 (emergency, hospital or registered physicians) and lay helpers in 2 (first aid drugs lay- or self-administered).

### Quality Management

At time of inclusion, centres received training to assure quality standards. An independent expert committee updated the questionnaire annually, based on e.g. double entry congruency. To account for heterogeneity of anaphylactic reactions, closed questions were complemented by free text manually.

Representativeness and accuracy of the anaphylaxis registry were assured through data collected directly from emergency physicians (EPs) in a sample catchment area. EPs were asked to complete a condensed version of the original questionnaire used for the Registry immediately after first aid treatment.

### Analysis

Independent evaluation was performed by two trained epidemiologists, using SAS 9.2 (SAS Institute Inc., Cary, U.S.). The cross-sectional information in the Registry allowed for the calculation of frequencies (of categories) only. Basic stratification was used to identify subgroup differences. Confidence intervals of prevalence measures were calculated using the standard Wald procedure. Weighting of EPs’ validation data was performed using a logistic regression model including age, cause and severity to match distribution of cases in anaphylaxis registry, Berlin catchment area. Missing data was minimized by individual queries involving the referral centres, if unavailable analyses were restricted to complete cases.

## Results

Of 83 referral centres participating in the anaphylaxis registry, 58 entered valid data between 2006 and 2010, 27 centres on 10 or more patients (17 dermatology, 9 paediatrics, 1 other). 2114 patients sought further evaluation of severe anaphylaxis, more than 85% within 6 months after the incident. In accord with population sizes, distribution of country membership (Germany 75.8%, Austria 9.1%, Switzerland 15.1%) and sex (female 47.2%) was well-balanced, with about one-fifth below legal age (20.2%). The most common assured cause of anaphylaxis was insect sting (47.9%), followed by food (16.0%) and drugs (9.4%, table 1).

**Table 1:** Patients in anaphylaxis registry, first aid treatment stratified by general characteristics and reaction circumstances.

			First aid treatment				
			Emergency physician	Physician in hospital	Physician in med. practice	Drugs lay-administered	Drugs self-administered
	n	(%)	%	%	%	%	%
**Total**	**2114**	(100.0)	**34.5**	**23.6**	**14.0**	**5.3**	**4.7**
**Country**							
Germany	**1602**	(75.8)	39.1	21.8	13.0	4.2	4.1
Austria	**193**	(9.1)	29.5	29.5	19.7	2.1	4.7
Switzerland	**319**	(15.1)	14.7	28.8	15.7	12.5	7.5
**Sex**							
Female	**998**	(47.2)	37.1	23.4	13.7	6.3	3.6
Male	**1116**	(52.8)	32.3	23.7	14.2	4.3	5.6
**Age**							
<18 years	**428**	(20.2)	20.8	26.6	15.2	18.7	3.0
18–64 years	**1401**	(66.3)	36.5	23.3	13.5	2.0	5.4
>64 years	**285**	(13.5)	45.3	20.7	14.7	1.1	3.9
**Location of anaphylactic reaction**							
Hospital, medical practice	**224**	(10.6)	5.4	48.7	29.5	1.3	0.9
Work place, school	**105**	(5.0)	34.3	22.9	14.3	10.5	2.9
Restaurant	**84**	(4.0)	33.3	29.8	2.4	8.3	9.5
At home	**524**	(24.8)	36.1	19.8	13.7	9.9	5.0
Outdoors (nature)	**452**	(21.4)	40.9	19.9	19.0	2.2	4.9
Outdoors (city)	**185**	(8.8)	44.3	19.5	10.3	6.5	5.4
**Cause (only confirmed)**							
Insect sting	**1012**	(47.9)	43.1	19.9	14.5	2.2	4.2
Food	**339**	(16.0)	24.8	21.5	10.3	16.5	6.2
Drugs	**198**	(9.4)	20.7	35.9	21.7	3.0	3.0
**Severity (following ** [**1**] **)**							
Severe reaction (II)	**587**	(27.8)	24.5	27.1	15.8	7.5	5.5
Shock (III)	**1448**	(68.5)	37.2	22.0	13.8	4.5	4.6
Resp./circulatory arrest (IV)	**64**	(3.0)	59.4	29.7	3.1	3.1	1.6

### First aid Treatment

One in three reactions were initially handled by an emergency physician (EP, 34.5%), 37.6% by other physicians, and 10.0% received first aid treatment through non-professionals (self- or lay-administered). However, Switzerland reported a lower frequency of EP treatment (14.7%) and a higher for lay-administered drugs (12.5%). Children and adolescents were also more likely to receive first aid treatment by lay helpers (18.7 vs. 1.9% in adults), especially at pre-school age (33.2%).

Of all anaphylactic reactions taking place in a hospital or medical practice (n = 224), 61.3% were caused by drugs and were most commonly treated by physicians on site (79.1%). Food items were responsible for 77.5% of severe anaphylaxis cases in restaurants (n = 84), and more often treated with self-administered first aid drugs than in other reactions (10.2 vs. 4.5%).

Severest cases with respiratory and/or circulatory arrest (n = 64) were more often treated by EPs (59.4 vs. 33.5%).

Recurrent disease accounted for 32.2% of registered reactions. Among these, first aid drugs were more commonly self- (13.1 vs. 1.6%) or lay-administered (8.5 vs 2.9%) compared to first occurrence cases.

### First Aid Drugs

**Figure 1:**
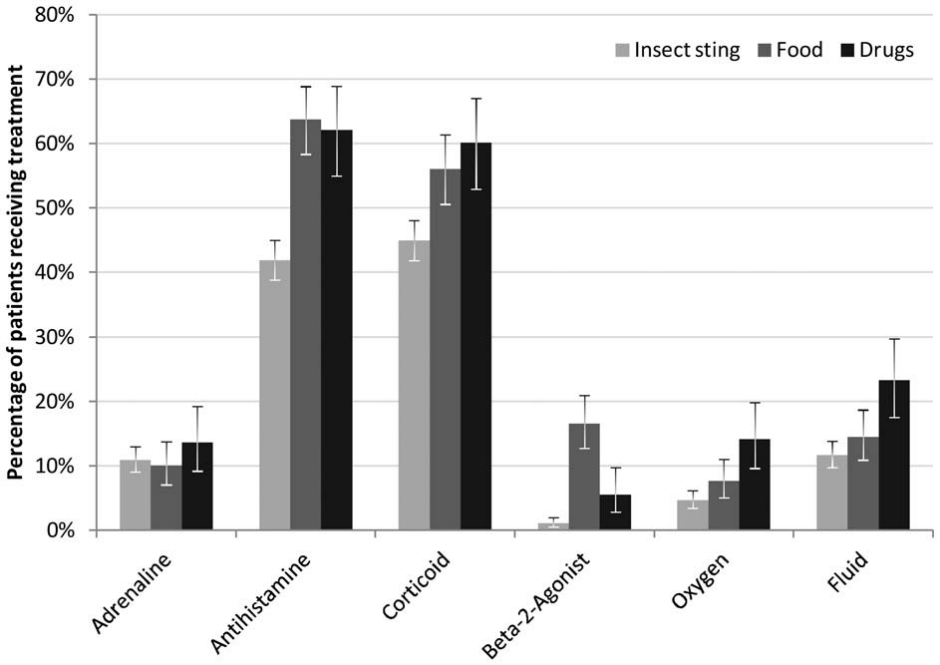
Drugs used for emergency treatment of anaphylaxis, by cause. Only assured cases. All application routes, error bars indicate 95% confidence intervals.

13.0% received adrenaline, irrespective of the cause. Application of antihistamines (50.1%) and corticoids (51.3%) was both less frequent in insect venom reactions compared to other elicitors. Beta-2-agonists were mainly given in food-induced anaphylaxis (5.9% in all reactions). Treatment with oxygen (6.3%) and fluids (13.9%) was more common in drug reactions than in insect venom or food-induced anaphylaxis (figure 1).

### Application Routes

**Figure 2:**
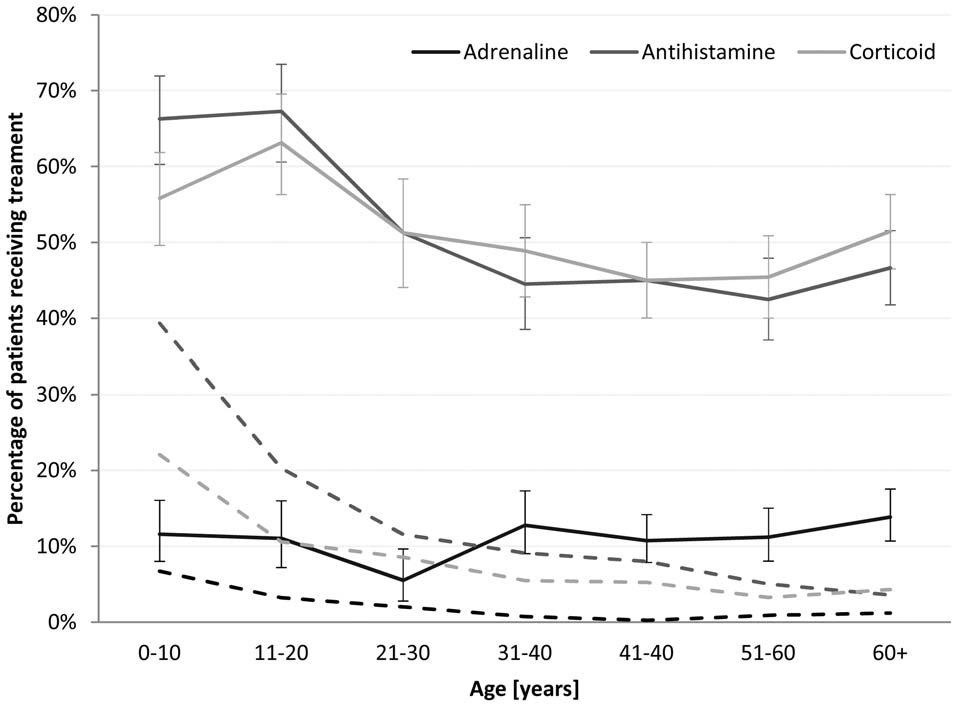
Drugs used for emergency treatment of anaphylaxis, by age. Dashed lines indicate proportion of patients having received inhalation (adrenaline) or oral (antihistamine, corticoid) treatment only, error bars indicate 95% confidence intervals.

Adrenaline was applied mainly intravenously (7.6%), compared to the recommended intramuscular route (3.9%), especially in Austria (18.7 vs. 3.1%). Only centres from Switzerland reported a frequent use of the intramuscular route (6.9%). EPs were more likely to apply adrenaline intravenously (11.9%), where self- and lay-administration was mainly intramuscular (23.2% and 12.6%). Less than half cases suffering respiratory and/or circulatory arrest received adrenaline (48.0%, table 2). With a steady age distribution for all application routes, adrenaline per inhalation was confined to the first 2 decades of life and decreased thereafter (figure 2).

**Table 2:** Application routes of drugs used for emergency treatment, stratified by country, first aid treatment and severity.

		Adrenaline			Antihistamine		Corticoid		Beta-2-Agonist	Oxygen	Fluid
		IM	IV	inhal.	IV	PO	IV	PO	inhal.		
	n	%	%	%	%	%	%	%	%	%	%
**Total**	**2114**	**3.9**	**7.6**	**2.3**	**38.1**	**13.8**	**42.9**	**8.7**	**5.9**	**6.3**	**13.9**
**Country**											
Germany	**1602**	3.4	6.4	1.9	34.9	11.0	40.8	6.1	5.5	5.7	12.2
Austria	**193**	3.1	18.7	2.6	54.4	6.7	58.5	2.6	3.6	11.4	32.6
Switzerland	**319**	6.9	6.6	4.1	44.2	31.7	44.2	25.4	9.1	6.3	11.0
**Inital treatment by**											
Emergency physician	**730**	1.5	11.9	2.6	48.1	3.7	54.0	2.5	2.7	8.5	17.8
Physician in hospital	**499**	3.4	7.8	2.8	51.3	10.0	57.7	5.4	7.0	6.6	15.6
Physician in med. practice	**296**	4.1	8.4	2.4	43.9	14.9	50.7	9.5	9.8	8.1	19.3
Drugs lay-administered	**111**	12.6	1.8	3.6	15.3	72.1	18.0	40.5	22.5	5.4	9.9
Drugs self-administered	**99**	23.2	1.0	0.0	6.1	66.7	6.1	53.5	8.1	1.0	1.0
**Severity (following ** [**1**] **)**											
Severe reaction (II)	**587**	4.3	2.7	2.2	38.5	19.4	43.6	12.1	10.2	3.6	6.1
Shock (III)	**1448**	3.9	8.0	2.1	38.0	11.9	42.5	7.5	4.4	6.4	16.4
Resp./circ. arrest (IV)	**64**	1.6	42.2	6.3	40.6	4.7	51.6	4.7	0.0	29.7	31.3

IM - intramuscular, IV - intravenous, PO – oral, inhal. - per inhalation.

Antihistamines and corticoids were given intravenously in most cases receiving that agent (38.1% and 42.9%), oral application was common in non-professional first aid treatment (table 2). Overall use of antihistamines and corticoids was highest in childhood and adolescence, mainly due to a high proportion of oral application, which declined in higher age groups (figure 2).

Inhalation of beta-2-agonists was confined to less severe reactions and most frequently lay-administered (22.5%).

Compared to other countries report of oxygen and fluid therapy was highest in Austria (11.4/32.6%). Only 29.7 and 31.3% of the most severe reactions (°IV) received oxygen and fluids (table 2).

### Validation Data

To account for reporting error in the anaphylaxis registry, we collected firsthand information on treatment of 218 severe anaphylaxis cases from EPs in the catchment area Berlin, Germany. Baseline characteristics were similarly distributed, except for an underrepresentation of females in the Anaphylaxis Registry (59.8 vs. 28.9%). Besides a lower frequency of drug-related anaphylaxis (18.8 vs. 7.8%) occurring mainly in hospitals (13.1 vs. 3.2%), reaction circumstances such as location, cause and severity recorded in the anaphylaxis registry resemble EP’s firsthand data (table 3).

**Table 3:** Baseline characteristics and reaction circumstances of severe anaphylaxis patients treated by emergency physicians.

	Emergency Physicians		Anaphylaxis Registry^#^			
	Berlin* (n = 218)		Berlin* (n = 90)		all centres (n = 730)	
	n	(%)	n	(%)	n	(%)
**Sex**						
Female	128	(59.8)	26	(28.9)	370	(50.7)
Male	86	(40.2)	64	(71.1)	360	(49.3)
**Age**						
<18 years	14	(6.4)	1	(1.1)	89	(12.2)
18–64 years	154	(70.6)	77	(85.6)	512	(70.1)
>64 years	50	(22.9)	12	(13.3)	129	(17.7)
**Location of anaphylactic reaction**						
Hospital, medical practice	28	(13.1)	2	(3.2)	12	(2.2)
Work place, school	11	(5.1)	6	(9.7)	36	(6.5)
Restaurant	6	(2.8)	10	(16.1)	28	(5.1)
At home	119	(55.6)	22	(35.5)	189	(34.2)
Outdoors (nature)	9	(4.2)	10	(16.1)	185	(33.5)
Outdoors (city)	34	(15.9)	9	(14.5)	82	(14.9)
**Cause (only confirmed)**						
Insect sting	49	(22.5)	23	(25.6)	436	(59.7)
Food	31	(14.2)	18	(20.0)	84	(11.5)
Drugs	41	(18.8)	7	(7.8)	41	(5.6)
**Severity (following ** [**1**] **)**						
Severe reaction (II)	33	(15.1)	25	(27.8)	144	(19.7)
Shock (III)	176	(80.7)	60	(66.7)	539	(73.8)
Resp./circulatory arrest (IV)	9	(4.1)	5	(5.6)	38	(5.2)

Reported by emergency physicians in Berlin, Germany vs. self report in anaphylaxis registry.

* comparable catchment areas, # only those initally treated by emergency physician.

**Figure 3:**
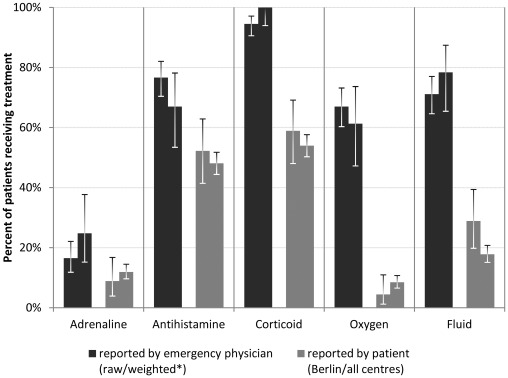
Drugs used by emergency physicians for initial treatment of anaphylaxis. Firsthand report (EPs) vs. self report (anaphylaxis registry). Parenteral application routes only. * Weighted for age, cause and severity distribution in anaphylaxis registry, Berlin catchment area.

Frequency of adrenaline administered parenterally was lower in the registry compared to Berlin EPs’ direct report, even after accounting for differences in baseline characteristics and reaction circumstances. Comparison of antihistamines and corticoids indicated a similar proportion of underreporting in the anaphylaxis registry. Failure to trace non-drug treatments became apparent matching data on oxygen and fluid therapy (figure 3).

### Instruction and Immunotherapy

61.5% of all cases were given general advice on anaphylaxis, including avoidance recommendation and delivery of allergy IDs. 75.1% received information on the use of first aid drugs in case of recurrent disease. Only a small proportion was instructed at the time of the initial reaction (11.6 and 19.6% respectively).

Children and adolescents were more likely to receive instructions (78.2/86.5%). Furthermore, cases of food-induced anaphylaxis were commonly given general and specific information on first aid drugs immediately following the incident (21.0/27.7%). 68.2/57.7% of patients with the most severe reactions (°IV) were instructed at any time (table 4).

**Table 4:** Patient instruction and specific immunotherapy after severe anaphylaxis, stratified by general characteristics and reaction circumstances.

		General instructions (avoidance, allergy ID)			Instructions for emergency medication			SIT
		at initial reaction	In between	at referral centre	at initial reaction	In between	at referral centre	
	n	%	%	%	%	%	%	%
**Total**	**2043**	**11.6**	**7.6**	**51.6**	**19.6**	**14.5**	**54.4**	**43.9**
**Children (<18 years)**	**410**	27.1	9.5	58.0	31.2	10.2	62.7	24.6
**Country**								
Germany	**1545**	12.9	8.6	47.8	18.6	17.2	51.7	45.6
Austria	**190**	3.2	5.3	53.2	7.9	8.4	61.1	50.5
Switzerland	**308**	10.7	3.9	69.8	31.5	5.2	63.6	31.2
**Type of referral centre**								
Dermatology	**1644**	7.9	7.2	49.1	17.2	15.8	53.4	48.4
Paediatrics	**320**	32.5	10.6	62.2	34.4	9.1	63.1	16.9
Internal medicine/ENT	**78**	5.1	2.6	60.3	9.0	10.3	39.7	60.3
**Cause (only confirmed)**								
Insect sting	**989**	8.9	9.2	45.0	21.7	20.0	60.6	82.6
Food	**328**	21.0	8.8	67.1	27.7	12.2	68.3	4.0
Drugs	**187**	16.0	5.9	79.1	6.4	3.2	22.5	0.5
**Most severe reactions (IV)**	**56**	17.9	3.6	58.9	16.1	12.5	39.3	37.5

SIT: Specific immunotherapy, ENT: Ear, nose and throat/Otolaryngology.

43.9% underwent specific immunotherapy, with the highest frequency in anaphylaxis caused by insect venom (82.6%).

### Prophylaxis

**Figure 4:**
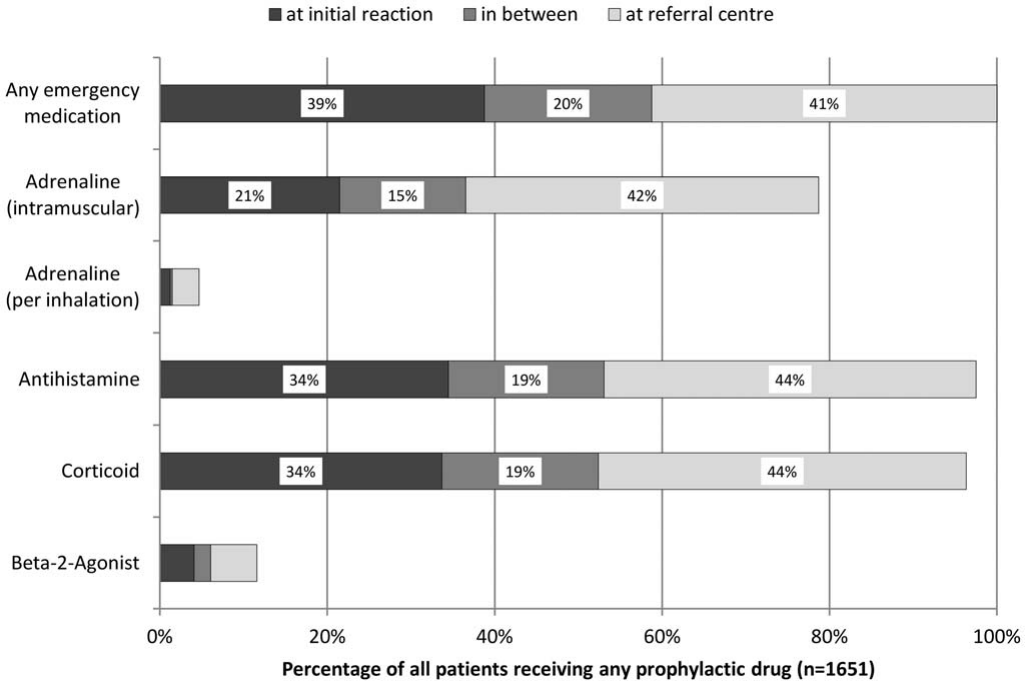
First time receiving prophylactic first aid drugs following severe anaphylaxis.

80.8% were given prophylactic first aid drugs at any time. Adrenaline auto-injectors were prescribed in 64.2%, oral antihistamines and corticoids in 79.1/78.0%. Of those receiving any prophylaxis, only one in five was given adrenaline and one in three antihistamines and corticoids immediately after the anaphylactic reaction (figure 4).

## Discussion

### Key Results

Severe anaphylaxis in the field was generally handled by professionals, involvement of lay helpers was only frequent in children and adolescents. Overall, less than 1 in 6 received adrenaline, even worse, only half of patients with respiratory or circulatory arrest. Though, application of antihistamines and corticoids was reported for the majority of anaphylactic reactions. Against current guidelines, adrenaline was applied intravenously by health professionals of any background in many cases.

Only 1 in 5 was provided with general information and adrenaline auto-injectors immediately after the incident, most patients received their first instructions at the referral centre visit. About 1 in 6 did not receive specific immunotherapy following severe insect venom anaphylaxis.

The distinct underuse of adrenaline is in line with several prior surveys of anaphylaxis management, for example from US emergency departments [18] or a questionnaire-based approach targeting German paediatricians [19]. The preference for intravenous application as seen in other settings (e.g. [20]) is not supported by guidelines or original literature [21], [22]. Yet, this analysis provides the first transnational and population-based survey of first aid treatment and secondary prevention of severe anaphylaxis. With a general perspective we demonstrated severe under- and misuse of adrenaline and failure to provide adequate patient instructions in the acute setting.

### Strengths and Weaknesses

All data in the anaphylaxis registry is derived from medical records in specialized referral centres, supplemented by emergency physician’s on-site documentation, if available. Transcription from the records is carried out by trained professionals and is shown to be accurate by double entry comparison. But content of medical records on the other hand is non-standardised, its integrity limited by the patient’s failure to spot and recall treatment details. Comparison with data collected directly from EPs fortunately revealed only a moderate underreporting of adrenaline, antihistamine and corticoid use in the anaphylaxis registry, not to the extent to change interpretation of the data. Only the use of oxygen and fluids is shown to be highly underreported.

To keep time and effort for the participating centres workable, we limited the set of items covered in the database and omitted several aspects such as dosing and timing of drugs or details of patient education. Patients were not traced prospectively to cover recidivism or impact of management modalities. Furthermore, our approach is generally limited to information conveyed through the patients, impeding statements concerning knowledge, degree of training or attitude towards different treatment options of the initial caregiver. For patients were identified at referral centres, this type of registry does not allow for inferring case fatality. However, up to date 6 deaths were reported to the registry via allergists.

Our target population comprises all individuals having experienced a severe anaphylactic reaction recently, living in a participating country. Our registry is not exhaustive as not all patients are referred to or follow the recommendation to present to a specialised allergy centre. Selection maybe influenced by socio-demographic background, perceived severity of anaphylaxis or other health-related attitudes. Furthermore, not all referral centres were traced or included in the study, rising concern about differential selection. Fortunately, general patient characteristics and circumstances of anaphylaxis based on data directly collected from EPs were comparable to our study population.

We assume to base the following interpretation on a highly standardized and valid set of primary data drawn from a sample representative of the general population.

### Implications

In light of established guidelines for the management of anaphylaxis, our survey confirmed known major gaps in their implementation on a transnational scale.

Failure to apply adrenaline timely and correctly in unquestionable severe anaphylaxis is most striking, in the field as well as in professional settings. In addition, only a minority of cases receive early and thorough patient education and preventive first aid drugs. We suppose a lack of knowledge and practical training to be responsible for these drawbacks. Our study identified not only EPs but all medical professionals as the target audience for continuing education, they are accountable for more than 90% of initial treatments, the main setting to advance management.

### Recommendations

To improve treatment of anaphylaxis, we strongly recommend revision of medical education and practical training, targeting a broad range of professionals [23]. This approach could foster a high coverage of guideline-conform management. We propose a close collaboration of physicians in primary care settings such as EPs and specialised allergists for the development of interventional strategies. With our strong data at hand, there is no reason to delay implementation of educational programs on a national or even transnational scale.

The European Academy of Allergy and Clinical Immunology (EAACI) is currently putting together an updated guideline for the management of anaphylaxis in children and adults (personal communication). Yet, to achieve sufficient implementation of current and future recommendations, a new approach for the dissemination of guidelines and continuing medical education is inevitable.

The future role of the anaphylaxis registry, currently embracing other European countries, is to monitor trends and evaluate interventional strategies aiming to improve patient care and other public health goals, concerning aspects of anaphylaxis occurrence, natural history of disease and management [24]. Alongside these given aims of our survey, comparison of treatment options might prove helpful to settle the longstanding debate about the effectiveness of first aid drugs, especially adrenaline, not yet resolved by interventional trials.
